# Acute abdomen with gastric volvulus revealing an underlying pneumatosis cystoides intestinalis: a case report

**DOI:** 10.1186/s12893-022-01717-6

**Published:** 2022-07-11

**Authors:** Mtanyous Chihab, Khaled Qadabashi, Huda Abbas, Maysam Attar, Ahmad Aljaber, Maden Alabd, Kusay Ayoub

**Affiliations:** 1 Department of Internal Medicine, Aleppo University Hospital, University of Aleppo, Aleppo, Syria; 2 Faculty of Medicine, University of Aleppo, Aleppo, Syria; 3 Department of General Surgery, Aleppo University Hospital, University of Aleppo, Aleppo, Syria

**Keywords:** Pneumatosis cystoides intestinalis, Gastric volvulus, Pyloric stenosis, Acute abdomen, Pneumoperitoneum

## Abstract

**Background:**

Pneumatosis intestinalis is an abnormal presence of free air outside the lumen of the intestines in many shapes. It is classified based on its etiology to primary or secondary, it affects adults as well as infants and can involve any part of the GI tract.

**Case presentation:**

We report a case of a 55-year-old man with a past medical history of a surgically repaired perforated duodenal ulcer who presented with an acute abdominal pain, Flatulence and constipation. On examination of the abdomen; severe distension, tenderness and tympanicity on percussion were noted. An erect CXR was performed and showed bilateral sub-diaphragmatic air levels. We performed an abdominal Paracentesis under the right subcostal margin which led to evacuation of large amounts of air. Next, an investigational laparotomy showed that the reason was a gastric volvulus associated with an anterior and posterior gastric wall lacerations. The suitable surgical repair approach was taken, but another lesion was detected incidentally. A pneumatosis cystoides intestinalis (PCI) was extended along large length of the intestines in many shapes and without any symptoms or signs.

**Conclusions:**

Pneumatosis cystoides intestinalis has been reported continuously in relation to peptic ulcer disease (PUD). We aim to report a new association of a gastric volvulus and PCI secondary to pyloric stenosis caused by a duodenal ulcer; which we believe can aid in the diagnosing of dangerous complications, of a rare disease.

## Background

Pneumatosis intestinalis (PI) is a condition where gas is accumulated inside or between the intestinal wall layers (Subserosa, Submucosa) [1]. PI can be classified as primary (15%) or secondary (85%). PI occurs in approximately 3% of population as the exact prevalence is still unknown [1, 2]. Computed tomography (CT) or endoscopy are usually used to detect PI. In primary PI the gas collection takes a cystic pattern whereas in secondary PI the gas appears in a more linear pattern [2]. The primary pattern of PI is termed pneumatosis cystoides intestinalis (PCI).

We present a case of an asymptomatic pneumatosis cystoides intestinalis which have been discovered along the entire length of the intestines incidentally by investigational laparotomy following acute abdominal presentation due to gastric volvulus complicated by tearing of the gastric wall.

An association between PCI and gastric volvulus was not found in the literature. We believe our case presents a rare presentation of an asymptomatic, complicated, incidentally discovered, widely spread PCI.

## Case presentation

A 55-year-old white male, with a past medical history of a surgically repaired perforated duodenal ulcer and no significant family medical history, presented to the emergency department of Aleppo University Hospital complaining of a 2-h period acute severe abdominal pain, flatulence, tachypnea and chest discomfort. The pain was sudden, dull aching in nature and periumbilical at first then quickly became generalized. The patient denied having any symptoms prior to presentation. On clinical examination, patient was alert, conscious, afebrile, tachypneic and distressed; with a pulse of 75 bpm and blood pressure of 140/90 mm Hg. His oxygen saturation was 96% in room air and ECG was normal. On examination of the abdomen, severe distension, tenderness and tympanicity on percussion were noted. Laboratory values included a normal white blood cell count and a normal serum urea nitrogen and creatinine levels, other serum laboratory values such as Na, K, Ca were measured to rule out electrolyte abnormalities and were within normal range.

**Fig. 1:**
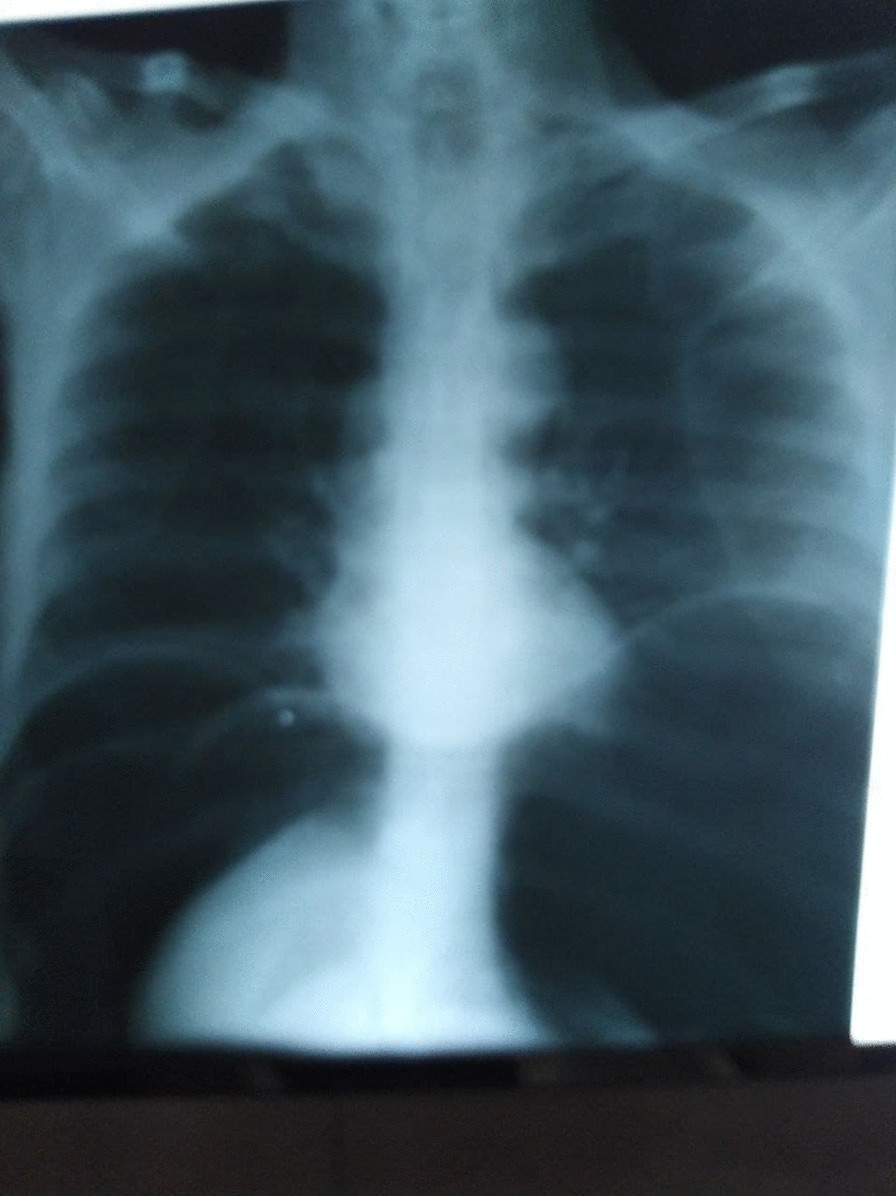
Plain upright PA-CXR. Image shows a large volume of sub-diaphragmatic free air

An erect CXR was performed and showed bilateral subdiaphragmatic air-levels [Fig. 1: the original resolution of this image is 1280 × 960, no downstream processing was applied]. Tension pneumoperitoneum was diagnosed-based on clinical picture, symptoms and radiographic features- and emergent paracentesis using a needle under the right costal margin was performed to reduce distention which led to evacuation of large amounts of air.

**Fig. 2:**
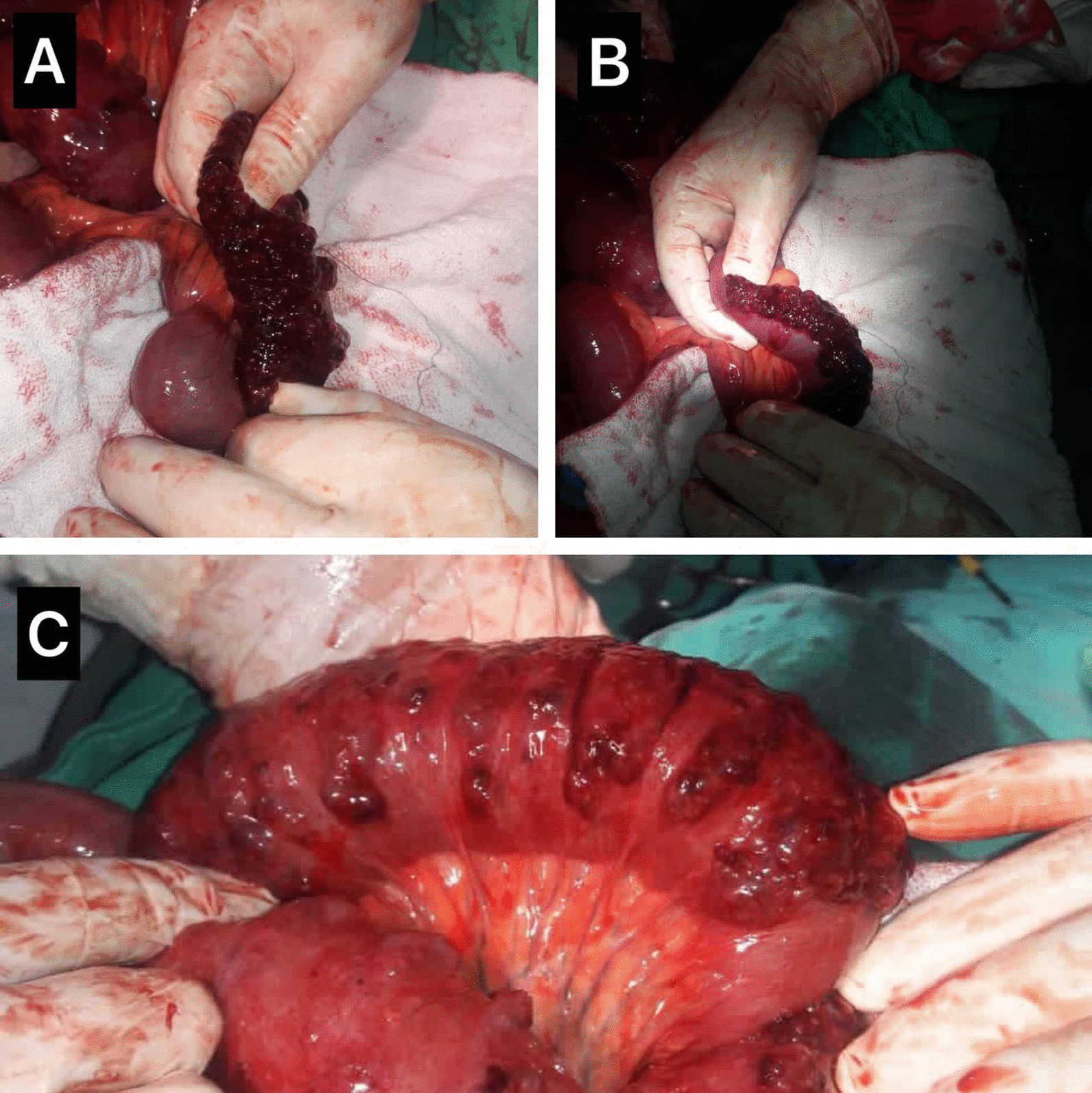
Gross appearance of affected loops. Image shows wide-spread, cystic lesions in the sub-serosa of small intestines

The patient was referred to surgery immediately-without performing a CT scan to avoid delay in management due to the urgent nature of the patient- and an emergency investigational laparotomy was performed through a standard midline incision over abdomen, stomach investigation revealed severe gastric distention, strangulated closed-loop gastric volvulus along with a full thickness, 4-cm long anterior and a 5 cm-long posterior gastric wall lacerations with a severely stenotic pylorus. Duodenum investigation revealed stenosis and fibrosis along the first part in site of an old ulcer. Small intestine investigation revealed multiple cystic lesions 140 cm away from Treitz ligament extending to large areas of small intestines [Fig. 2: the original resolution of this image is 1280 × 960, no downstream processing was applied], the lesions were sub-serosal, bubble-like, fragile and necrotic in some areas. Some degree of ischemia was found along the intestines that can be attributed-based on clinical picture, massive pneumoperitoneum and diffuse ischemic changes of bowels- to abdominal compartment syndrome. Surgical repair of the gastric lacerations and a gastro-jejunal anastomosis were performed to bypass the stenosis. Due to the extensive spreading of PCI and ischemia of the bowels and the absence of localized necrosis, surgical resection of such long length of bowel was not considered.

**Fig. 3:**
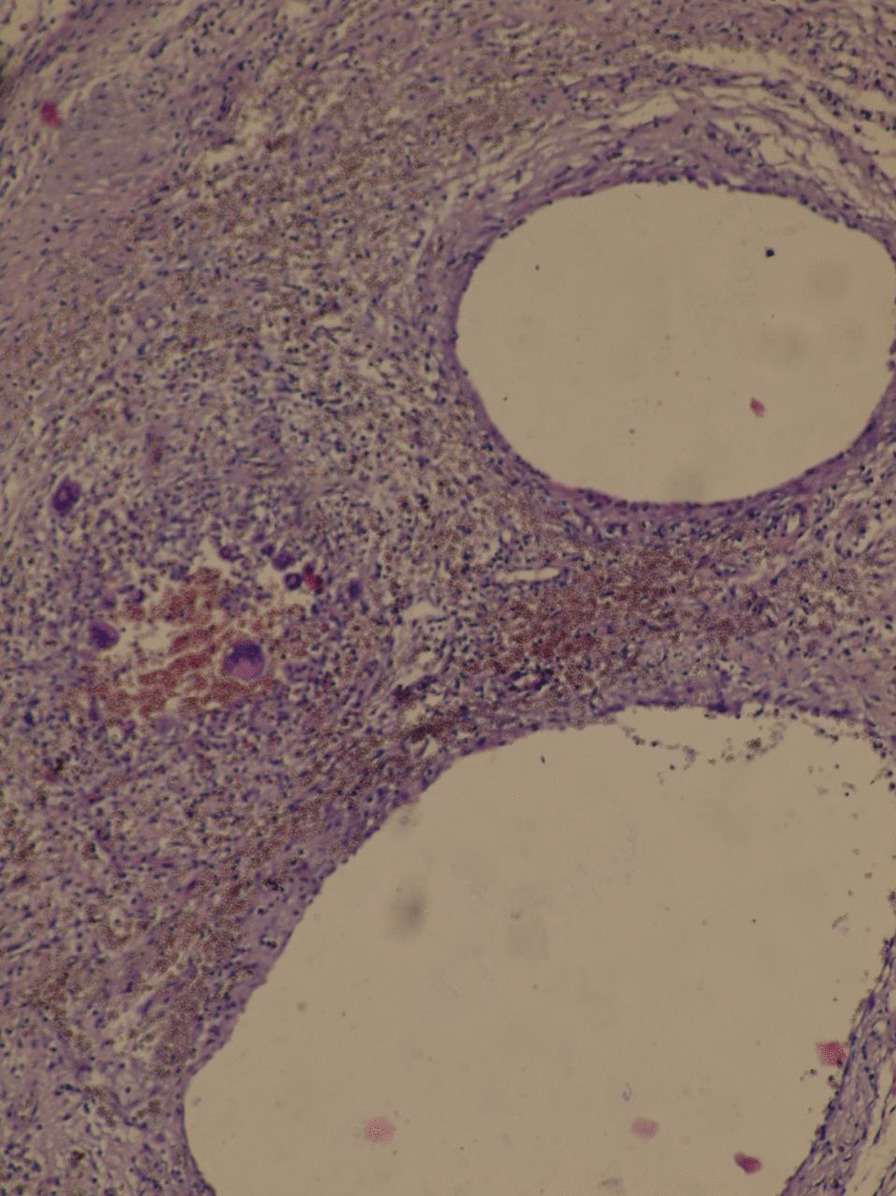
Microscopic image of incisional biopsy. Image shows chronic inflammatory reaction characterized by histiocytes and giant cells lining multiple cysts (This image was taken by Olympus OM 35 mm SLR microscope, original magnification, × 40; scale bar, 100 µm)

**Fig. 4:**
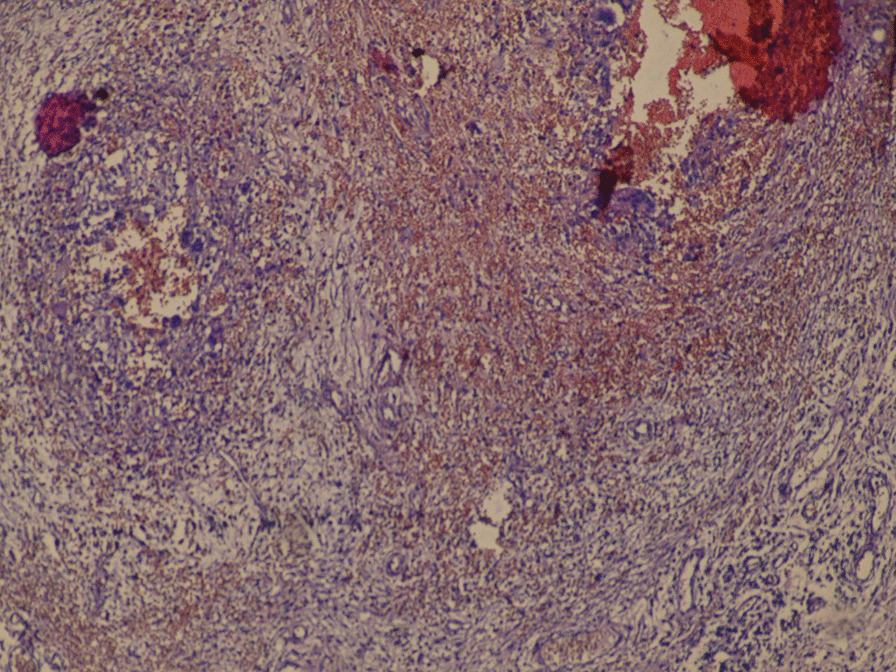
Microscopic image of incisional biopsy. Image shows chronic inflammatory reaction characterized by histiocytes and giant cells lining multiple cysts (This image was taken by Olympus OM 35 mm SLR microscope, original magnification, × 100; scale bar, 100 µm)

Biopsies were taken from cystic lesions, and revealed cysts with focal granulomas composed of aggregated epithelioid histiocytes and multiple foreign body-type multinucleated giant cells surrounding the cysts [Figs. 3, 4: the original resolution of this images is 1280 × 960, no downstream processing was applied].

Post-operatively; over the next 48 h, the patient became hypotensive, tachycardic and developed altered mental status, hypoxia [Spo2 = 82%], fever and leukocytosis [WBC 15.8] with neutrophilia. The patient was shortly intubated and broad-spectrum antibiotics were initiated immediately along with fluids and vasopressors but unfortunately the patient died soon afterwards. The presumed cause of death is probably either due to ischemia–reperfusion injury and reactive oxygen species or septic shock. Post-mortem pathology and/or autopsy was not performed due to refusal of family to consent.

## Discussion and conclusions

Pneumatosis intestinalis refers to gas accumulation inside the intestinal wall in different forms and causes with poorly understood etiology [3, 4]. It can affect any part of the GI tract but large intestines are more commonly affected [5, 6]. Sex predominance has been controversial, in a retrospective review of PCI, Koss [7] and Jamart [8] found a higher incidence in males whereas a prospective study by Knechtle et al. [9] showed equal incidence among males and females. Primary forms are more likely to be asymptomatic and discovered incidentally whereas secondary forms are multifactorial and more likely to be symptomatic and complicated [3].

This case shows a rare presentation of PCI. Over time, an obstruction of stomach outlet lead to a gastric volvulus which was complicated by gastric wall tearing and presented as an acute abdomen. This condition required investigational laparotomy which lead to the discovery of intestinal PCI.

PI is usually defined by endoscopy or CT scan of the abdomen, secondary to gastrointestinal illnesses or other common diseases such as COPD, diabetes mellitus and connective tissue diseases, … etc. [5, 10]. PI is so rare to be idiopathic and asymptomatic even wide spreading [11].

Multiple theories regarding explanation of PCI were introduced in the literature, including mechanical, pulmonary, bacterial and biochemical theories [12]. The mechanical theory is the most compatible with our case; Two main mechanisms are used to explain this theory: mucosal injury and increased intraluminal pressure. Perforated duodenal ulcer causes injury to the mucosa, which in turn allows gas to enter through layers of the intestinal wall. And the pyloric stenosis causes increased intraluminal pressures which forces gas to escape the lumen and settle in the subserosa.

Our patient had a past history of a surgically repaired duodenal ulcer; PUD was mentioned in the literature as a common cause of PCI [12] and numerous papers reported an association between PCI and gastric outlet obstruction (pyloric stenosis) [12, 13]. In our case the stenosis was accompanied by a gastric volvulus and complicated by tearing of the anterior and posterior gastric walls; which is a new, unprecedent clinical scenario related to PCI.

Most of PI cases are secondary to other diseases which makes them easier to detect in the context of the primary illness. On the other hand, PCI can be asymptomatic which means that the diagnosis is discovered either incidentally or because of its complications. Complications are rare in PI with 3% incidence rate, but being serious conditions makes diagnosis the earlier, the better. Most common ones are pneumoperitoneum, intestinal obstruction and perforation, volvulus, and intestinal ischemia [4, 14–16].

Association between PI and volvulus is rare. One case report published in 2021 showed a pneumatosis intestinalis associated with a rare volvulus proximal to the ileocecal junction [17], Gillon et al. published a report of four cases of PI associated with sigmoid volvulus [18]. While in our case it was associated with a gastric volvulus complicated by two lacerations and led to a life-threatening situation. In the previously mentioned reports, the presence of volvulus was explained by PCI locally acting as a starting point of volvulus. While in our case, the absence of PCI in the pylorus does not explain PCI as the cause. In this paper we describe a dyad of gastric volvulus and PCI with unclear causation; and to our knowledge, no previous similar dyad has been described before.

A systematic review, published in 2013, showed that PI appeared more commonly in the colon, followed by the small intestine, followed by both of them combined in a really small number of cases [4]. Our case shows an extensive PCI that spreads along large areas of the intestines but without symptoms or signs until complications happened. Complications could be so harsh especially in a patient with no single specific symptom and a wide-spreading lesion.

According to a retrospective study by Morris et al. [6], half of patients were successfully treated non-operatively; conservative treatment includes using hyperbaric oxygen therapy or metronidazole, while third were managed operatively. Our patient on the other hand was diagnosed incidentally, he was referred to surgery immediately due to acute presentation and presence of tension pneumoperitoneum which dominated the clinical picture. IV metronidazole was added to the antibiotic regimen, but hyperbaric oxygen therapy was not considered due to the unavailability of hyperbaric chambers.

In conclusion, PCI can be caused by multiple diseases including peptic ulcer disease, and it can be benign and managed conservatively, or present as a dangerous life-threatening complication that requires intervention. Knowledge of the possible complications is crucial in the diagnosis of such rare, but potentially devastating condition.

## Data Availability

The datasets used and/or analyzed during the current study are not publicly available due to privacy and ethical restrictions but are available from the corresponding author on reasonable request.

## Abbreviations

PI: Pneumatosis intestinalis
PCI: Pneumatosis cystoides intestinalis
CXR: Chest X-ray
WBC: White blood cells
GI: Gastrointestinal
CT: Computerized Tomography
COPD: Chronic Obstructive Pulmonary Disease
PUD: Peptic ulcer disease
